# Apomorphine formulation may influence subcutaneous complications from continuous subcutaneous apomorphine infusion in Parkinson’s disease

**DOI:** 10.1007/s00415-020-10031-1

**Published:** 2020-07-01

**Authors:** Peter Hagell, Arja Höglund, Carina Hellqvist, Eva-Lena Johansson, Berit Löwed, Anne-Christine Sjöström, Carina Karlberg, Margareth Lundgren, Nil Dizdar, Anders Johansson, Thomas Willows, Johan Rådberg, Filip Bergquist

**Affiliations:** 1 The PRO-CARE Group, Faculty of Health Sciences, Kristianstad University, 291 88 Kristianstad, Sweden; 2 Department of Neurology, Karolinska University Hospital, Stockholm, Sweden; 3 Department of Neurology, University Hospital, Linköping, Sweden; 4 Department of Neurology, Karlstad Central Hospital, Karlstad, Sweden; 5 Department of Neurology, Sahlgrenska University Hospital, Gothenburg, Sweden; 6 Department of Biomedical and Clinical Science, Linköping University, Linköping, Sweden

**Keywords:** Apomorphine, Complications, Nodules, Parkinson’s disease, Safety, Skin

## Abstract

Continuous subcutaneous (s.c.) apomorphine infusion is an effective therapy for Parkinson’s disease (PD), but a limitation is the formation of troublesome s.c. nodules. Various chemically non-identical apomorphine formulations are available. Anecdotal experiences have suggested that shifting from one of these (Apo-Go PumpFill^®^; apoGPF) to another (Apomorphine PharmSwed^®^; apoPS) may influence the occurrence and severity of s.c. nodules. We, therefore, followed 15 people with advanced PD (median PD-duration, 15 years; median “off”-phase Hoehn and Yahr, IV) on apoGPF and with troublesome s.c. nodules who were switched to apoPS. Data were collected at baseline, at the time of switching, and at a median of 1, 2.5, and 7.3 months post-switch. Total nodule numbers (*P* < 0.001), size (*P* < 0.001), consistency (*P* < 0.001), skin changes (*P* = 0.058), and pain (*P* ≤ 0.032) improved over the observation period. PD severity and dyskinesias tended to improve and increase, respectively. Apomorphine doses were stable, but levodopa doses increased by 100 mg/day. Patient-reported apomorphine efficacy tended to increase and all participants remained on apoPS throughout the observation period; with the main patient-reported reason being improved nodules. These observations suggest that patients with s.c. nodules caused by apoGPF may benefit from switching to apoPS in terms of s.c. nodule occurrence and severity. Alternatively, observed benefits may have been due to the switch itself. As nodule formation is a limiting factor in apomorphine treatment, a controlled prospective study comparing local tolerance with different formulations is warranted.

## Introduction

Apomorphine is a highly potent dopamine receptor agonist whose clinical antiparkinsonian effects first were reported in 1951 [1]. It has been used in the treatment of Parkinson’s disease (PD) since the late 1980s, either as intermittent subcutaneous (s.c.) injections or as continuous s.c. apomorphine infusion (CSAI) [2–4]. The clinical antiparkinsonian symptomatic efficacy of CSAI is similar to that of other advanced therapeutic options for PD (e.g., deep brain stimulation and levodopa/carbidopa intestinal gel) [5] and has similar cost-effectiveness [6]. However, a limiting factor is the relatively frequent occurrence of troublesome s.c. nodules at infusion sites. For example, a review of clinical apomorphine studies found s.c. nodules to occur in more than 70% of people treated with CSAI [3], and in a recent randomized double-blind placebo-controlled trial, it was reported in 44% of cases in the active arm at 3 months, while it did not occur with placebo [7].

Although it is relatively uncommon that s.c. nodules lead to discontinuation of therapy, they can be troublesome and associated with itching or soreness, and sometimes pain, skin discolouration and scarring; in severe cases, ulceration and necrosis may also occur [4, 8]. Furthermore, nodules may lead to suboptimal s.c. apomorphine uptake with reduced clinical benefit [8, 9]. Attention to skin hygiene, rotation of infusion sites, massage and ultrasound at infusion sites are recommended to reduce s.c. nodules [8, 10–14], but does not eliminate the problem.

The mechanisms behind the development of s.c. nodules are still not fully understood. However, histological studies demonstrate eosinophilic infiltration and suggest cell-mediated allergic type IV hypersensitivity or late phase type I response to apomorphine or its solution additives [15–17]. There is also some evidence that higher apomorphine concentrations and rapid auto-oxidation of apomorphine may increase s.c. nodule formation [18, 19].

There are various apomorphine formulations with non-identical chemical properties available. It is, therefore, plausible that these may differ with respect to s.c. nodule formation. Indeed, unpublished anecdotal clinical experience from Sweden has suggested that shifting from one of these (Apo-Go Pumpfill^®^ 5 mg/ml; apoGPF, manufactured in the UK/Belgium/Spain/Germany) to another (Apomorfin PharmSwed^®^ 5 mg/ml; apoPS, manufactured in Sweden)^1^ may influence the occurrence and severity of s.c. nodules. Therefore, we undertook a systematic clinical observational audit of CSAI-treated people with PD who experienced troublesome s.c. nodules to explore if the occurrence and severity of s.c. nodules appear to be influenced by the pharmaceutical formulation of apomorphine used for s.c. infusion. Our primary objective was to assess whether the number and size of nodules changed following a shift from apoGPF to apoPS, but also to monitor other nodule-associated features and clinical PD-related factors.

## Methods

This prospective observational pragmatic clinical audit was conducted at four Swedish PD centres (three university hospitals and one regional hospital). Fifteen consecutive CSAI-treated people with advanced PD and with troublesome s.c. nodules were switched from apoGPF to apoPS. Management of existing nodules (ultrasound, massage, etc.) and other CSAI-related aspects (e.g., dressings, infusion site hygiene) continued as per clinical routine, as were any adjustments of apomorphine doses and other drugs. All participants provided informed consent.

Data were collected between 2015 and 2017; at baseline, at the time of switching (median (min–max), 1.9 (0.1–12.9) weeks later), and at regular clinical follow-up visits at 0.8–1.5 (median, 1), 1.7–4.2 (median, 2.5), and 5.3–11.9 (median, 7.3) months post-switch. Patient assessments were based on clinical routine at the participating centres, and included demographic data; Hoehn and Yahr stage of PD [20] as assessed from clinical observations and patient-reported history for the past week; patient-reported overall PD severity (rated 1–3, representing “mild”, “moderate” and “severe” [21]); the Unified PD Rating Scale (UPDRS [22]) part I (items 1–4; total score, 0–16; higher scores = worse) to assess neuropsychiatric symptoms; and UPDRS part IV to assess dyskinesias (items 32–35; total score, 0–13; higher scores = worse) and motor fluctuations (items 36–39; total score, 0–7; higher scores = worse). Ongoing PD therapy was recorded and expressed as daily levodopa equivalent doses (LED; mg) [23]. All assessments concerned the week immediately before and including the day of the visit.

A s.c. nodule assessment protocol was used to assess the number and size of s.c. nodules, as well as associated potential nodule-related features. The nodule assessment protocol was based on routine clinical assessments and recommendations from the literature [8], and included: total number of s.c. nodules; nodule diameter (mm; as assessed using a standard pair of callipers); nodule consistency (rated 0–3, representing “none”, “soft”, “medium”, and “hard”, respectively); visible nodule-associated skin changes (rated 0–4, representing “none”, “light redness”, “obvious redness”, “pronounced redness” and “ulceration”, respectively); average nodule-associated pain during the last week, unprovoked nodule pain and palpation induced tenderness at the time of assessment. Average and current pain or tenderness was rated 0–5, representing “none”, “mild”, “discomforting”, “distressing”, “horrible” and “excruciating”, respectively [24]. Patient-reported reliability and efficacy of the apomorphine effect during the past week was also assessed (rated 0–4, representing “very poor” through “very good”). Assessments of nodule size, consistency, associated skin changes and current pain were limited to the five worst nodules.

At the last follow-up, patients were asked whether they considered switching back to apoGPF, and the reason(s) for doing or not doing so. Those not available for clinical assessment were contacted by telephone.

Data were analyzed using Stata version 15.1 (StataCorp, College Station, TX). Due to the ordinal nature and generally non-normal distributional properties of data, non-parametric statistics were used. Comparisons across all five timepoints were made according to the Skillings-Mack test and explorative pairwise comparisons between timepoints were done using the Wilcoxon signed-ranks test. *P* values < 0.05 were considered statistically significant. Due to the small sample size and the exploratory nature of our observations, we did not correct for multiple comparisons.

## Results

Baseline sample characteristics are summarized in Table 1. Participants had been diagnosed with PD since a median of 15 years and had been on waking day CSAI treatment for 2.2 years. Median Hoehn and Yahr stages of PD were II during “on” and IV during “off”. All but one used supplemental bolus doses of apomorphine (0.65–23.7 mg/day) in addition to the continuous infusion (22.8–156.5 mg/day) and four used intermittent s.c. apomorphine injections (10 mg/ml; 7.5–14 mg/day), primarily in the morning to enable preparation and start of the apomorphine infusion pump. Other antiparkinsonian therapies consisted of immediate (*n* = 14) and controlled-release (*n* = 3) levodopa/decarboxylase inhibitor, entacapone (*n* = 7), rasagiline (*n* = 7), rotigotine (*n* = 4), pramipexole (*n* = 3), amantadine (*n* = 2), and ropinirole (*n* = 1). Domperidone was used by one individual and quetiapine by another. The majority (*n* = 13; 87%) was using one or several interventions to treat existing s.c. nodules: massage (*n* = 10), ultrasound (*n* = 5), and glucosaminoglycan polysulfate (Hirudoid^®^) cream (*n* = 2). With few exceptions, these were used daily or almost daily.

**Table 1:** Baseline sample characteristics (*n* = 15)

Characteristics	
Gender, *n* (%)	
Male	10 (67)
Female	5 (33)
Age, median (q1–q3; min–max)	66 (64–71; 55–74)
Years since PD diagnosis, median (q1–q3; min–max)	15 (8.8–16.8; 5.6–23)
Years on CSAI, median (q1–q3; min–max)	2.2 (0.7–2.5; 0.2–8.8)
Marital status, *n* (%)	
(Living as) married	14 (93)
Single	1 (7)
Educational level, *n* (%)	
Comprehensive school	4 (27)
Upper secondary school	4 (27)
University	7 (47)
Living condition, *n* (%)	
Own home	14 (93)
Nursing home/assisted living	1 (7)
“On”-phase Hoehn and Yahr stage of PD, *n* (%)	
Stage I (unilateral)	3 (20)
Stage II (bilateral)	6 (40)
Stage III (postural instability)	3 (20)
Stage IV (severe)	2 (13)
Stage V (unable to stand or walk)	1 (7)
“Off”-phase Hoehn and Yahr stage of PD, *n* (%)	
Stage I (unilateral)	0 (0)
Stage II (bilateral)	0 (0)
Stage III (postural instability)	3 (20)
Stage IV (severe)	8 (53)
Stage V (unable to stand or walk)	4 (27)
Ongoing nodule intervention(s), *n* (%)	13 (87)

*PD* Parkinson’s disease, *CSAI* continuous subcutaneous apomorphine infusion

**Fig. 1:**
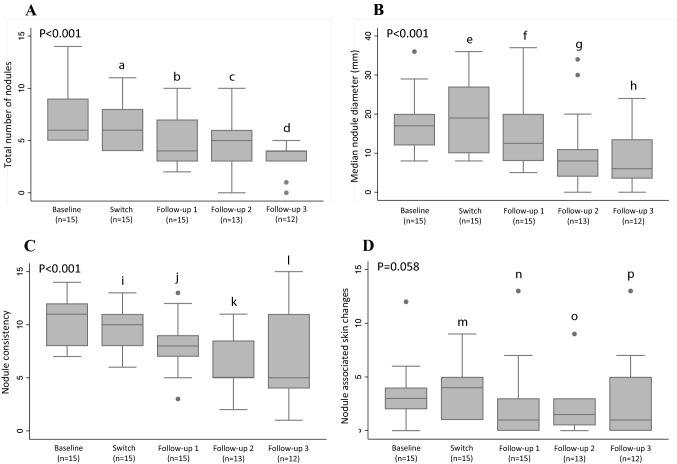
Development of subcutaneous (s.c.) nodules over time in relation to switching from one apomorphine formulation (Apo-Go Pumpfill^®^ 5 mg/ml) to another (Apomorfin PharmSwed^®^ 5 mg/ml) among 15 people on continuous s.c. apomorphine infusion with troublesome s.c. nodules. **a** Depicts the total number of s.c. nodules at the various time points; **b** through **d** depict the (**b**) median nodule diameter (mm) and total sums of ratings of nodule (**c**) consistency (rated 0–3; 0 = none, 3 = hard) and **d** associated skin changes (rated 0–4; 0 = none, 4 = ulceration) of the five most pronounced nodules for each individual at the respective time points. *P* values in the respective panels are for comparisons (Skillings-Mack tests) across all time points. Pairwise comparisons (Wilcoxon signed-ranks tests) yielded the following results: (a) *P* = 0.042 vs baseline; (b) *P* = 0.011 vs. baseline, *P* = 0.030 vs. switch; (c) *P* = 0.002 vs baseline, *P* = 0.033 vs. switch, *P* = 0.497 vs. follow-up 1; (d) *P* = 0.002 vs baseline, *P* = 0.003 vs. switch, *P* = 0.053 vs. f ollow-up 1, *P* = 0.232 vs. follow-up 2; (e) *P* = 0.755 vs baseline; (f) *P* = 0.004 vs. baseline, *P* = 0.002 vs. switch; (g) *P* = 0.016 vs baseline, *P* = 0.009 vs. switch, *P* = 0.133 vs. follow-up 1; (h) *P* = 0.002 vs baseline, *P* = 0.003 vs. switch, *P* = 0.041 vs. follow-up 1, *P* = 0.724 vs. follow-up 2; (i) *P* = 0.261 vs baseline; (j) *P* = 0.019 vs. baseline, *P* = 0.008 vs. switch; (k) *P* = 0.002 vs baseline, *P* = 0.006 vs. switch, *P* = 0.112 vs. follow-up 1; (l) *P* = 0.040 vs baseline, *P* = 0.082 vs. switch, *P* = 0.373 vs. follow-up 1, *P* = 0.964 vs. follow-up 2; (m) *P* = 0.167 vs baseline; (n) *P* = 0.142 vs. baseline, *P* = 0.136 vs. switch; (o) *P* = 0.217 vs baseline, *P* = 0.124 vs. switch, *P* = 0.249 vs. follow-up 1; (p) *P* = 0.370 vs baseline, *P* = 0.245 vs. switch, *P* = 0.298 vs. follow-up 1, and *P* = 0.341 vs. follow-up 2. Solid horizontal lines are median values, boxes are inter-quartile ranges (25th–75th percentiles), error bars are ranges, and dots are outliers

Results from assessments at the various time points are presented in Fig. 1 and Table 2. At baseline, participants had between 5 and 14 s.c. nodules (median, 6). This number decreased following the switch of apomorphine formulation to a median of 4 at the third follow-up 5–12 months later and disappeared altogether in one case (Fig. 1). Before the switch, the total median diameter of the five most pronounced nodules was 78 mm, which decreased throughout the observation period to 32.5 mm at the final follow-up. In parallel, nodule consistency as well as nodule-associated skin changes improved (Fig. 1), although the latter failed to reach statistical significance. The number of cases (*n* = 13) using interventions to treat existing nodules was stable up until the first post-switch follow-up but decreased to 9 and 8 at the second and third follow-ups, respectively. Nodule-associated pain and tenderness decreased following the switch, whereas the number of needle changes per day was relatively stable over the observation period (Table 2).

**Table 2:** Outcomes over time in relation to switching from one apomorphine formulation (Apo-Go Pumpfill^®^ 5 mg/ml) to another (Apomorfin PharmSwed^®^ 5 mg/ml) among 15 people on continuous s.c. apomorphine infusion with troublesome s.c. nodules

	Baseline (*n* = 15)	Switch (*n* = 15)	Follow-up 1 (*n* = 15)	Follow-up 2 (*n* = 13)	Follow-up 3 (*n* = 12)	*P* value^b^
Average pain during past week^c^	1 (0–2; 0–4)	1 (0–1; 0–3)	0 (0–1; 0–3)	0 (0–0; 0–1)^j,k,l^	0 (0–0; 0–2)^m^	0.008
Unprovoked current pain^c,d^	0 (0–2; 0–5)	0 (0–0; 0–1)^j^	0 (0–0; 0–1)^j^	0 (0–0; 0–1)^q^	0 (0–0; 0–4)^r^	0.028
Tenderness to palpation^c,d^	0 (0–4; 0–9)	2 (0–4; 0–8)	0 (0–2; 0–4)^m,n^	0 (0–2; 0–5)^k,q^	0 (0–1; 0–6)^r^	0.032
Number of needle changes/day	2 (1–2; 1–4)	2 (1–3; 1–4)	2 (1–2; 0–3)^m,n^	2 (1–2; 1–3)	2 (1–2; 1–3)	0.128
“Off”-phase Hoehn and Yahr stage	IV (IV–V; III–V)	IV (IV–V; III–V)	IV (III–V; II–V)^n^	IV (III–IV.5; II–V)^n^	IV.5 (III–V; II–V)	0.099
“On”-phase Hoehn and Yahr stage	II (II–III; I–V)	III (II–III; I–V)	III (II–III; I–V)	III (II–III; I–V)	II (II–III; I–V)	0.410
Patient-reported PD severity^e^	3 (2–3; 2–3)	2 (2–2; 1–3)^j^	2 (2–3; 1–3)	2 (2–3; 1–3)	2 (2–3; 2–3)^k^	0.091
Neuropsychiatric symptoms, UPDRS part I^f^	1 (1–3; 0–10)	1 (0–3; 0–10)	1 (0–5; 0–8)	2 (0–4.5; 0–6)	1.5 (0–6; 0–9)	0.834
Dyskinesias, UPDRS part IV^g^	2 (1–5; 0–6)	2 (1–4; 0–8)	3 (1–4; 0–4)	3 (1–4; 0–5)^k^	3.5 (1–4; 0–9)^n,l^	0.130
Motor fluctuations, UPDRS part IV^h^	4 (3–5; 2–7)	4 (2–4; 1–5) ^j^	3 (3–5; 2–6)	3 (2–5; 1–6)^j^	4 (2–5; 0–6)	0.066
Patient-reported apomorphine reliability^i^	2 (1–3; 0–4)	2 (2–3; 0–4)	3 (1–4; 0–4)	3 (2–3; 1–4)	3 (2–3; 1–4)	0.467
Patient-reported apomorphine efficacy^i^	2 (2–4; 0–4)	3 (2–4; 1–4)	3 (3–4; 2–4)^m^	3 (2–3.5; 2–4)^o^	3 (2–4; 2–4)^k^	0.143
Daily total LED (mg)	1633 (1230–1958.5; 1140–2394)	1661 (1240–1963; 1062.5–2394)	1620.5 (1240.5–1963; 1076.5–2394)	1595 (1248–1899.5; 1066.5–2157.5)	1712 (1345–1987; 1081.5–2853.5)^m,n,l,p^	0.005
Daily apomorphine LED (mg)	980.5 (710–1228; 353–1802)	955.5 (710–1228; 290–1942)	980.5 (710–1198; 240–1942)	916.5 (635–1211.5; 240–1340.5)	1006 (678.5–1313; 401.5–1455)	0.786
Daily levodopa LED (mg)	400 (250–800; 75–1330)	400 (266–800; 0–1330)	400 (200–800; 0–1330)	400 (200–785.5; 0–1000)	500 (249–748; 0–1413.5)^j,n,l,p^	0.002
Daily total LED (mg) excluding apomorphine	640 (430–906; 175–1330)	620 (400–906; 175–1380)	620 (366–906; 175–1380)	620 (415–862; 200–1580)	680 (391.5–907; 200–1580)^j,n,l,p^	0.004

Data are median (q1–q3; min–max). Pairwise *P* values ≤ 0.1 (Wilcoxon signed-ranks tests) are indicated

*PD* Parkinson’s disease, *UPDRS* Unified Parkinson’s disease Rating Scale, *LED* levodopa equivalent dose

^b^Skillings-Mack test

^c^Rated 0–5 (0 = none, 5 = excruciating)

^d^Total score from the 5 most pronounced nodules

^e^Rated 1–3 (1 = mild, 2 = moderate, 3 = severe)

^f^Possible score range, 0–16 (higher scores = more neuropsychiatric symptoms)

^g^Possible score range, 0–13 (higher scores = more dyskinesias)

^h^Possible score range, 0–7 (higher scores = more motor fluctuations)

^i^Rated 0–4 (0 = very poor, 4 = very good)

^j^*P* < 0.05 vs. baseline

^k^*P* = 0.05–0.10 vs. switch

^l^*P* < 0.05 vs. follow-up 1

^m^*P* = 0.05–0.10 vs. baseline

^n^*P* < 0.05 vs. switch

^o^P = 0.05–0.10 vs. follow-up 1

^p^P < 0.05 vs. follow-up 2

^q^*n* = 12

^r^*n* = 11

Clinical PD-related features were generally stable and did not change significantly over the observation period (Table 2). However, there was a slight improvement in “off”-phase Hoehn and Yahr following the switch, and there was a trend towards increased dyskinesias over the follow-up period. There were also slight improvements in patient-reported overall PD severity and motor fluctuations between baseline and time of switching (Table 2).

Daily apomorphine doses were stable throughout the observation period, but total daily LED showed a slight increase at the final follow-up, mainly driven by levodopa (Table 2). At the final follow-up, continuous apomorphine infusion doses ranged between 27 and 145.5 mg/day, with additional bolus doses of 0.95–14.75 mg/day; two participants remained on s.c. apomorphine injections in the morning (12–18 mg/day). Alterations in other dopamine agonists, amantadine, domperidone and quetiapine therapies were marginal and non-significant (data not shown).

There was a non-significant trend for improved patient-reported apomorphine efficiency following the switch (Table 2) and all participants remained on apoPS throughout the observation period. Patient-reported reasons for not switching back to apoGPF included fewer or improved s.c. nodules (*n* = 10), more user-friendly device (*n* = 3), less nodule-related pain (*n* = 1), better apomorphine efficacy (*n* = 1), and general treatment satisfaction (*n* = 2).

## Discussion

Our observations in a series of 15 people with PD who were experiencing troublesome s.c. nodule formations due to CSAI therapy suggest that switching from one apomorpine formulation (apoGPF) to another (apoPS) can improve the number, size and consistency of nodules, as well as nodule-associated pain. This improvement occurred within the first month post-switch and remained for up to a year under stable daily apomorphine doses and decreased use of interventions to treat existing s.c. nodules. Although not directly comparable, these findings appear to suggest better outcomes than previously reported for treatment with ultrasound [14].

The mechanism(s) behind our observations remain speculative. One pharmacological factor that may influence nodule formation is the apomorphine concentration [18]. However, apoPS and apoGPF both have the same concentration. Differences between the two apomorphine formulations, as specified in the respective summaries of product characteristics,^2^ include sodium chloride, sodium, sodium metabisulfite and pH, and may thus (independently or in interaction) be involved in the improvement of s.c. nodules observed here. Alternatively, the switch itself (i.e., the mere fact that there is a change in tissue exposure) may contribute to a decreased propensity for nodule formation. That is, it cannot be ruled out that a switch from apoPS to apoGPF also may be beneficial in cases of s.c. nodules developed under treatment with apoPS.

PD symptoms and severity as well as total daily apomorphine doses were largely stable over the observation period. However, there was a slight improvement in “off”-phase Hoehn and Yahr and a slight increase in dyskinesias following the switch. This could be explained by a simultaneous increase in total daily dopaminergic treatment, mainly represented by an increased oral levodopa dose of about 100 mg. However, there was also a tendency for patients to perceive a somewhat improved apomorphine efficacy. This suggests that an alternative explanation could be an improved apomorphine efficacy secondary to reduced s.c. nodule formation and subsequent increased apomorphine bioavailability.

Our observations are primarily limited by a small sample size together with its pragmatic observational nature without a control group and with relatively large variability in the timing of assessment points. This latter variability was due to individual variations in clinical follow-up, since the protocol did not prescribe exact follow-up times but was based on clinical praxis. However, a pragmatic observational approach such as this may reflect clinical reality better than a well-controlled study with strict protocol adherence. There was a decrease in the number (but not size) of nodules from baseline to switch, which could suggest a regression-to-the-mean effect. However, the numbers (5–14) and sizes (8–36 mm) of nodules at baseline cover fairly large ranges and do not represent a particular subgroup, apart from the fact that their nodules were considered problematic. Furthermore, there was a continuous decrease in both numbers and size over the subsequent timepoints, which is in contrast to previous observations on untreated or sham ultrasound treated CSAI-associated nodules [14]. This argues against a predominant regression-to-the-mean effect. We did not correct for multiple comparisons in our statistical analyses. This is a small series of exploratory observations and any findings would have to be confirmed in a larger controlled study anyway. However, when applying the Bonferroni correction (a critical P-value of 0.0025 for an alpha level of 0.05), improvements of the main variables (i.e., the number, size and consistency of nodules) remained statistically significant. The fact that we observed statistically significant changes despite a small sample with additional attrition over time, suggests that the observed effects are not trivial. Nevertheless, there is a need for larger, controlled studies for firm conclusions and to determine if improvement only occurs with switches from apoGPF to apoPS or if tolerability to different formulations is an individual characteristic.

With these limitations in mind, our observations suggest that patients with s.c. nodules caused by apoGPF may benefit from switching to apoPS in terms of s.c. nodule occurrence and severity, and that skin tolerance is related to factors in the drug formulation other than the apomorphine concentration. If these observations can be confirmed, there is a possibility to improve the clinical acceptability of this modality of advanced PD therapy.

## Footnotes

1 Because of identical generic drug names, we use the respective Swedish trade names, which differ somewhat across countries (apoGPF and apoPS are, e.g., available as APO-go 5 mg/ml oplossing voor infusie in een voorgevulde spuit and Apomorfine hydrochloride PharmSwed 5 mg/ml, oplossing voor infusie, respectively, in the Netherlands).

2 See https://www.medicines.org.uk/emc/medicine/15992 (apoGPF) and https://docetp.mpa.se/LMF/Apomorfin%20PharmSwed%20solution%20for%20infusion%20ENG%20SmPC_09001be6803dcd2a.pdf (apoPS) for summaries of product characteristics.

## References

[1] Schwab RS, Amador LV, Lettvin JY (1951). Apomorphine in Parkinson’s disease. Trans Am Neurol Assoc.

[2] Stibe CMH, Kempster PA, Lees AJ, Stern GM (1988). Subcutaneous apomorphine in parkinsonian on-off oscillations. Lancet.

[3] Deleu D, Hanssens Y, Northway MG (2004). Subcutaneous apomorphine: an evidence-based review of its use in Parkinson’s disease. Drugs Aging.

[4] Hagell P, Odin P (2014). Apomorphine in Parkinson’s disease.

[5] Dafsari HS, Martinez-Martin P, Rizos A, Trost M, dos Santos Ghilardi MG, Reddy P, Sauerbier A, Petry-Schmelzer JN, Kramberger M, Borgemeester RWK, Barbe MT, Ashkan K, Silverdale M, Evans J, Odin P, Fonoff ET, Fink GR, Henriksen T, Ebersbach G, Pirtošek Z, Visser-Vandewalle V, Antonini A, Timmermann L, Ray Chaudhuri K (2019). EuroInf 2: subthalamic stimulation, apomorphine, and levodopa infusion in Parkinson’s disease. Mov Disord.

[6] Walter E, Odin P (2015). Cost-effectiveness of continuous subcutaneous apomorphine in the treatment of Parkinson’s disease in the UK and Germany. J Med Econ.

[7] Katzenschlager R, Poewe W, Rascol O, Trenkwalder C, Deuschl G, Chaudhuri KR, Henriksen T, van Laar T, Spivey K, Vel S, Staines H, Lees A (2018). Apomorphine subcutaneous infusion in patients with Parkinson’s disease with persistent motor fluctuations (TOLEDO): a multicentre, double-blind, randomised, placebo-controlled trial. Lancet Neurol.

[8] Poltawski L, Edwards H, Todd A, Watson T, James C-A, Lees A (2008). Cutaneous side effects of infused apomorphine: the patient and carer experience. Br J Neurosci Nurs.

[9] Lees A, Turner K (2002). Apomorphine for Parkinson’s disease. Pract Neurol.

[10] Trenkwalder C, Chaudhuri KR, García Ruiz PJ, LeWitt P, Katzenschlager R, Sixel-Döring F, Henriksen T, Sesar Á, Poewe W, Baker M, Ceballos-Baumann A, Günther D, Drapier S, Ebersbach G, Evans A, Fernandez H, Isaacson S, van Laar T, Lees A, Lewis S, Castrillo JCM, Martinez-Martin P, Odin P, O’Sullivan J, Tagaris G, Wenzel K (2015). Expert Consensus Group report on the use of apomorphine in the treatment of Parkinson’s disease—clinical practice recommendations. Park Relat Disord.

[11] Pietz K, Hagell P, Odin P (1998). Subcutaneous apomorphine in late stage Parkinson’s disease: a long term follow up. J Neurol Neurosurg Psychiatry.

[12] McGee P (2002). Apomorphine treatment: a nurse’s perspective. Adv Clin Neurosci Rehabil.

[13] Todd A, James CA (2008). Apomorphine nodules in Parkinson’s disease: best practice considerations. Br J Community Nurs.

[14] Poltawski L, Edwards H, Todd A, Watson T, Lees A, James CA (2009). Ultrasound treatment of cutaneous side-effects of infused apomorphine: a randomized controlled pilot study. Mov Disord.

[15] Acland KM, Churchyard A, Fletcher CL, Turner K, Lees A, Dowd PM (1998). Panniculitis in association with apomorphine infusion. Br J Dermatol.

[16] Van Laar T, Van Hilten B, Neef C, Rutgers AWF, Pavel S, Bruijn A (1998). The role of EDTA in provoking allergic reactions to subcutaneous infusion of apomorphine in patients with Parkinson’s disease: a histologic study. Mov Disord.

[17] Borgemeester RWK, van Laar T, Schuttelaar MLA (2018). Cutaneous adverse drug reaction after apomorphine infusion, possibly caused by a systemic type IV hypersensitivity reaction to sodium metabisulfite: report of 2 cases. Contact Dermatitis.

[18] Borgemeester RWK, Diercks GFH, Jonkman MF, Van Laar T (2015). Treatment of apomorphine-induced skin reactions: a pilot study. Mov Disord.

[19] Burgemeester RWK, Diercks GFH, Schuttelaar MLA, Van Laar T (2016). Histology of subcutaneous nodules in PD patients treated with continuous apomorphine infusion: case series and review of the literature. Mov Disord.

[20] Hoehn MM, Yahr MD (1967). Parkinsonism: onset, progression and mortality. Neurology.

[21] Hagell P, Sandlund B (2000). Patients’ self-assessment of disease and symptom severity in Parkinson’s disease. Qual Life Res.

[22] Fahn S, Elton RL, Fahn S, Marsden CD, Calne DB, Goldstein M, committee members of the U development (1987). Unified Parkinson’s Disease Rating Scale. Recent developments in Parkinson’s Disease.

[23] Tomlinson CL, Stowe R, Patel S, Rick C, Gray R, Clarke CE (2010). Systematic review of levodopa dose equivalency reporting in Parkinson’s disease. Mov Disord.

[24] Melzack R (1975). The McGill Pain Questionnaire: major properties and scoring methods. Pain.

